# Practicing What We Preach: Investigating the Role of Social Support in Sport Psychologists’ Well-Being

**DOI:** 10.3389/fpsyg.2015.01854

**Published:** 2015-12-15

**Authors:** Hannah M. McCormack, Tadhg E. MacIntyre, Deirdre O’Shea, Mark J. Campbell, Eric R. Igou

**Affiliations:** ^1^Department of Physical Education and Sport Sciences, University of Limerick, Limerick, Ireland; ^2^Department of Employment Relations, Kemmy Business School, University of Limerick, Limerick, Ireland; ^3^Department of Psychology, University of Limerick, Limerick, Ireland

**Keywords:** self care, work engagement, burnout, sport psychology, ethics, social support, mental health, qualitative

## Abstract

Well-being and mental health of psychologists and their clients can be strongly linked to the psychologists’ experience of work. We know from general theories of occupational health psychology that certain work factors will have a greater impact on well-being than others. Work engagement is positively related with occupational health, while burnout and workaholic tendencies relate negatively. An individual’s resources can buffer against these negative effects. Specifically, the environmental resource of social support can impede the impact and instance of workaholism and has a positive influence on burnout. Social support is often encouraged by sport psychologists in protecting an athlete’s well-being. Drawing on theory and research from work and organizational, health and social psychology we explore the lived experiences of burnout and work engagement among applied sport psychologists, investigating their perceptions of how these experiences impact their well-being. Thirty participants from five countries were asked, using semi-structured interviews, to recall specific incidents when feelings of work engagement and burnout occurred. We examined the influence of social support and its impact on these incidents. Thematic analysis revealed that burnout is frequently experienced despite high levels of work engagement. Sources of social support differ between groups of high burnout versus low burnout, as does reference to the dimensions of work engagement. Avenues for future research including investigating the role of mindfulness and therapeutic lifestyle changes for practitioners are outlined.

## Introduction

Increasingly, scrutiny is being exerted on the application of psychology across a range of settings. Applied psychology, comprising the fields of clinical, health, forensic, sport and exercise, occupational and educational psychology all have a common challenge of solving personal and social problems associated with human behavior (Davey, 2011). Our study investigated a specific aspect of applied psychology, namely sport psychology, in order to elucidate practitioner well-being and the challenges therein. Firstly, it is worthwhile to explore the broader context in which our practitioners must operate; the nuances of the evolving field of applied sport psychology will be discussed later.

Among the reasons for accountability in our profession are the financial pressures on healthcare systems and the necessity to demonstrate positive client outcomes (APA, 2006), the emergence of clear educational pathways for practitioner psychologists (e.g., sport psychology; Fletcher and Maher, 2013) and the development of competency benchmarks across applied disciplines (Fouad et al., 2009). One step toward meeting the demands for the professionalization of the discipline has been the development of statutory regulation, which, for example, has been in situ in the UK since 2009 (Health Care Professionals Council; HCPC). The role of this agency is to protect the public by ensuring that applied psychologists meet specified standards of training, behavior and professional skills and as a consequence are “fit to practice.” Under this system practitioners have “a personal responsibility to maintain and manage [one’s] own fitness to practice and are required to engage in self-referral if changes to health and character may impede the ability to practice” (HPC, 2007). Critically, this approach focuses on an individual’s competencies without considering their social context (e.g., working as part of a multidisciplinary team). Furthermore, practitioner self-care is largely overlooked in the standards and the question remains: who will protect the practitioner? This is not a moot point given the potential for compassion fatigue, secondary traumatic stress (Figley, 2002), burnout and mental health challenges among practitioners (Malinowski, 2014). In addition to practitioner well-being issues, the client may be at risk and so, self-care is an ethical imperative (Barnett et al., 2007).

### The Social Context of Sport Psychology

Applied sport psychology is a rapidly growing profession (Arnold and Sarkar, 2014; Campbell and Moran, 2014) that addresses issues central to sport and physical performance, deals with sportsmen, sportswomen and associated professionals, and requires knowledge of factors that can facilitate and enhance sporting performance (Andersen et al., 2001). More recently, Andersen and Speed (2010) argued that the primary role of a sport psychologist was the welfare of the client rather than simply focusing on enhancing performance. Thus, the psychologist may share the collaborative goal of seeking performance enhancement, while recognizing the influence of goal achievement on the well-being of the client.

This juxtaposition of meeting the goals of performance enhancement and well-being are not new to the field of sport psychology. Historically, sport psychology grew up in physical education departments, subsequently termed “sport-science” or “kinesiology” faculties (Andersen et al., 2001). Consequently, client mental health and well-being was not typically at the forefront of the interventions, which instead focused upon performance enhancement using psychological skills training programs. This psycho-educational approach did not dilute the application of a more comprehensive psychological approach entirely as the field was still influenced by the 1965 Boulder scientist-practitioner model. In fact, one of the most common interventions in the early years of modern sport psychology was developed by a clinical psychologist for use in sport settings (e.g., visuo-motor behavioral rehearsal, Suinn, 1997). In recent years, the commonality between clinicians and sport psychologists has gained precedence. Emerging evidence has suggested that the prevalence of mental health challenges among sporting populations are at least as common as among the non-sporting samples (Schaal et al., 2011). This challenges the assumption of the prototypical model in the field, the mental health model for sport (Raglin, 2001), which simply linked training load to mental health challenges rather than the myriad of issues that may occur with the social context of a sporting sub-culture (e.g., risk of eating disorders in esthetic sports; Brewer and Petrie, 2014). Consequently, the requirement for more comprehensive training in mental health for neophyte practitioners is now clearer than ever.

Practitioner self-regulation is of particular interest to sports psychology because of the potential challenges with regard to managing multiple relationships (including boundaries and dual agency), the potential for isolation, overcoming clients protective nature (Brown et al., 2005) and disparate training routes that consultants have pursued that may not have provided training in specific competencies for self-care and peer support (Aoyagi and Portenga, 2010). One common example is how practitioners operate at the Olympic Games (Birrer et al., 2012). Over 3 weeks of the competition, they typically interact with athletes in non-traditional time segments and locations, which may involve multiple roles, exhaustive time commitments, isolation from family and friends, and potential client goal conflict (Andersen et al., 2001). In this environment the burden of ethical behavior often rests solely with the practitioner and it is essential that they remain self-aware and self-regulating in order to remain a benefit to their clients and ultimately themselves (Haberl and Peterson, 2006).

Some of the aforementioned challenges may resonate with clinical psychologists and a number of these issues have been highlighted by researchers in mental health and ethics (Koocher and Keith-Spiegel, 2007). Service delivery in the sporting context can occur during both formal (e.g., at training) and informal settings (e.g., on the bus to the event) therefore practitioners can themselves feel under pressure to consistently perform (McCann, 2008). The expectation to consistently provide a service is arguably a case of applied psychology *in extremis* and provides a rationale for our current study, which focuses upon the practitioner as a performer as well as a service provider (Fletcher et al., 2011). This continual pressure to perform, managing not only the performance goals of the athlete, may result in burnout in the long term.

### Burnout as a Risk

Prevailing research in the domain of work and organizational psychology explains the psychological and social factors in determining mental health in the workplace. Building on Karasek’s (1979) job-demand’s control model, Bakker and Demerouti (2007) introduced the job demands-resources model, which incorporated psychological resources, job resources and job demands as considerations in understanding burnout and work engagement (Demerouti et al., 2001). This model posits two fairly independent processes, a health impairment process—associated with an end state of burnout—and a motivational process—associated with work engagement (Bakker et al., 2014). In this context, *burnout* refers a state of exhaustion and cynicism toward work (Bakker et al., 2014), which is conceptualized as a psychological syndrome in response to chronic interpersonal stressors on the job (Maslach et al., 2001). It has three key dimensions: *exhaustion* refers to feelings of being overextended and depleted in one’s psychological resources, *cynicism* or *depersonalization* represents the interpersonal context of burnout, and refers to negative and detached responses to aspects of the job, and *reduced efficacy* or accomplishment is a self-evaluation referring to feelings of incompetence and reduced productivity (Maslach et al., 2001).

A longitudinal study conducted by Sonnentag et al. (2010) found that high demands matched with low psychological detachment, where the individual is unable to stop thinking about work during non-work times, was associated with increased reports of emotional exhaustion in the long term. A lack of detachment can increase strain, which negatively affects resource attainment and management, and on a daily basis can negatively influence and individual’s approach to work the following day (Sonnentag, 2012). It has also been suggested that increased strain on individuals, fortified through low levels of psychological detachment, resulted in increased reports of psychosomatic complaints (Sonnentag, 2012).

We adopt the perspective that clinical settings are a specific type of work context, with specific demands and resources, and thus, it is appropriate to adopt work-based theories, rather than general clinical models, in seeking to explain burnout for those who work in clinical settings. From such a perspective, the type of work engaged in by sports psychologists working with athletes is very similar to the person-focused work of medical professions such as doctors and nurses. The job demands-resources model has been applied to such professions, demonstrating high levels of burnout due to the intensive demands imposed by caring for others in need, and particularly due to the high levels of emotional labor that are required (Demerouti et al., 2000, 2009; Brotheridge and Grandey, 2002). For example, Dunford et al. (2012) examined burnout across career transitions. Their research demonstrated that the three burnout dimensions differ in their pattern of change over time as a result of career transition type: organizational newcomers, internal job changers (e.g., promotions or lateral moves), and organizational insiders (i.e., job incumbents). Using a large sample of health care employees, over 2 years, they found that burnout was relatively stable for organizational insiders but slightly dynamic for organizational newcomers and internal job changers. In addition, they found that the dimensions of emotional exhaustion and depersonalization were more sensitive to career transition type than reduced personal accomplishment.

Such research is of particular relevance to sport psychologists, where they frequently operate across multiple organizational boundaries, and may hold multiple role identities (Andersen et al., 2001). Similarly, while they may form part of the support function with high performance teams or athletes, their role as a psychologist may mean that they remain somewhat of an “outsider” operating in a professional vacuum. Aoyagi and Portenga (2010) describe this as a role “in which the practitioner is the only person in the environment with knowledge of professional roles, responsibilities, and ethics” (p. 258). Ambiguity resulting from managing multiple role identities is a well-established cause of stress and burnout. For example, research has demonstrated that workers distinguish between organizational, workgroup and career foci of identification (Millward and Haslam, 2013), that there are circumstances when work identity is negatively associated with well-being (Avanzi et al., 2012), and that managing multiple roles is associated with stress and burnout (Rothbard et al., 2005). Critical outcomes of burnout are decreased job performance and increased absenteeism (see Bakker et al., 2014, for a review). Furthermore, burnout has been found to be contagious and thus, there is evidence that experiences of burnout can be transferred to others in contact with the burned out individual (Bakker et al., 2005). For psychologists in clinical settings, such outcomes will undoubtedly impact on their client interaction and capacity to support them.

Contemporary research has introduced interpersonal strain as an additional dimension of burnout (Consiglio, 2014). Interpersonal strain represents “the feeling of discomfort and disengagement in the relationships with people at work resulting from exceeding social requests and pressures” (Borgogni et al., 2011, p. 875). Research has established its distinctiveness from established burnout dimensions (Borgogni et al., 2011) and it has been shown to be related to emotional dissonance and health symptoms in hospital staff (Consiglio, 2014). As healthcare professionals, psychologists are also subject to the effects of high levels of interpersonal and emotional demands, which may result in high levels of experienced interpersonal strain. Thus, like any other healthcare professional, psychologists may be particularly susceptible to burnout. However, in contrast to other healthcare professionals, psychologists are trained in self-care techniques for managing psychological health and well-being. Interestingly, there may be expectations that practitioners are expected to be able to manage their mental health appropriately in themselves. Thus, there may be a degree of stigma associated with burnout for a psychologist and this could reduce their engagement with processes such as self-referral. Clients, employees, colleagues, and even family and friends may be perceived as questioning the abilities of a psychologist who is struggling with their own psychological distress (Barnett et al., 2007). However, Bearse et al. (2013) reported that although stigma is not rated highly as a barrier toward seeking personal psychotherapy, almost two thirds of respondents admitted to not seeking psychotherapy at a given time even though they recognized that it could have benefited them. Bearse et al. (2013) suggest that this could be related to the perceived privacy issues associated with visiting another psychologist, such as being seen as a client of someone else which could deter an applied practitioner from seeking the desirable help.

### Work Engagement as a Resource

The job demands-resources model posits that the experience of work engagement is an antidote to burnout (e.g., Gonzalez-Roma et al., 2006; Schaufeli et al., 2008). In this case, work engagement represents a state of mind characterized by feelings of vigor, dedication and absorption (Schaufeli et al., 2002). Vigor is characterized by high levels of energy and resilience while working, the willingness to invest effort in one’s work and persistence in the face of difficulties. Dedication is characterized by a sense of significance, enthusiasm, inspiration, pride, and challenge (Schaufeli et al., 2002). Finally, absorption is characterized by being fully concentrated and deeply engrossed in one’s work, whereby time passes quickly and one has difficulties in detaching oneself from work (Schaufeli et al., 2002). Vigor and dedication are considered direct opposites of the exhaustion and cynicism components of burnout (Bakker et al., 2014). Work engagement has been shown to be related to decreases in ill-health, and an increase in job performance and life-satisfaction (Shimazu et al., 2015).

Accumulating evidence demonstrates strong support for the proposition that highly engaged employees are much less likely to experience burnout, and even experience fatigue in a different way (Schaufeli et al., 2006). “Engaged employees have a sense of energetic and effective connection with their work activities, and they see themselves as able to deal well with the demands of their jobs” (Schaufeli et al., 2006, p. 702). Moreover, work engagement is fostered when job and personal resources meet the demands faced in the job (Bakker and Demerouti, 2008). Although high workload and multiple roles are associated with burnout as discussed above, there are situations when the negative effects of such job demands may be mitigated and engagement can still be experienced. For example, Hakanen et al. (2008) demonstrated that dentists engagement was not affected by high workload when they experienced high skill variety. However, when they experienced low skill variety, engagement decreased as a function of increasing qualitative workload. Thus, engagement may contribute the upward spirals of resource gain (Salanova et al., 2010), which can buffer against the negative health impairment spiral of resource loss that can lead to burnout.

### The Role of Social Support as a Resource

Prevailing research suggests that social support is one of the most important job resources in combating burnout and facilitating engagement, and has been the most extensively studied job resource in buffering against burnout (Maslach et al., 2001; Halbesleben and Buckley, 2006; Blanch and Aluja, 2012). “Social support” refers to an individual’s belief that help is available from other people in different situations (Cobb, 1976; Mayo et al., 2012). Recent research on interpersonal strain has utilized the conservation of resources theory (Hobfoll, 1989) to account for the relationship between the social environment and burnout. The basis of this theory is that people have a drive to create, foster, conserve, and protect the quality and quantity of their resources (Gorgievski and Hobfoll, 2008). Burnout, from this perspective, is a stress outcome resulting from a process of the slow bleed out of resources without any counterbalancing resource gain or replenishment (Gorgievski and Hobfoll, 2008). Utilizing this perspective, social support has been found to be a job resource that buffers the effect of stress (Cohen and Wills, 1985; Bakker et al., 2004; Mayo et al., 2012) and thus should ameliorate the onset of burnout. Social support has also been shown to be inversely related to burnout in a sporting context (DeFreese and Smith, 2013). Experimental research suggests that replenishment occurs given the occurrence of favorable conditions (Tyler and Burns, 2008). From this perspective, social support may be a mechanism through which burned out individuals try to create such favorable conditions.

The picture may be somewhat more complex than this, however. One key area of investigation lies in examining the source of social support, which may be work-related (e.g., supervisor, co-workers) or non-work-related (e.g., family; Halbesleben, 2006), and vary in terms of whether it is formal (e.g., counseling service) or informal. A meta-analysis of sources of social support and burnout (Halbesleben, 2006) found that the existence of social support as a resource, did not demonstrate relationships across any of the three burnout dimensions. However, when considering the sources of social support, a different view emerged. Work-related sources of social support were more closely associated with exhaustion than depersonalization or personal accomplishment, while the opposite pattern was found with non-work sources of support (Halbesleben, 2006).

### The Present Study

Social support is encouraged by sport psychologists in the maintenance of an athlete’s well-being (DeFreese and Smith, 2014). Despite the extensive literature establishing that social support is an important job resource in combatting burnout, little research has examined the lived experience of burnout for those who are meant to be experts in its management. In this study, we aimed to explore the experience of burnout for sport psychologists using a qualitative approach. Specifically, we were interested in the lived experience of managing burnout in professionals who “should know better” and the cognitive dissonance associated with both managing athletes’ psychological health and well-being, while simultaneously experiencing burnout themselves.

Secondly, we aimed to encapsulate work engagement and its sources among sport psychologists, specifically examining the different aspects of work engagement and how these differed between those who were deemed to have experienced either low or high levels of burnout.

Finally we captured critical incidents of burnout experienced by sport psychologists, and qualitatively examined whether those with experienced high or low levels of burnout cited different sources of social support, including formal versus informal, and work-related versus non work-related sources of social support.

## Materials and Methods

### Participants

A total of 51 participants provided informed consent to participate in an online survey, from a target sample of 80, from which 30 agreed to participate in follow-up interviews. Purposive sampling was employed to obtain a comprehensive exploration of the topics among an international sample of sport psychology practitioners (limited to Anglophone countries, i.e., the USA, the UK, Ireland, Australia, and New Zealand). Inclusion criteria were as follows: (a) practitioners had to be currently accredited or certified as a sport psychologist by a relevant organization [i.e., Association of Applied Sport Psychology (AASP), HCPC, Australian Psychological Society, or Irish Institute of Sport] and (b) they must also work within the high performance environment (have attended an international competition such as the Olympics or Paralympics, World Cup, European Cup, Pan-American or the Commonwealth Games in the role of sport psychologist or have worked with athletes who have competed at this level). Institutional ethical approval was granted by the Education and Health Sciences Faculty Research Committee for all aspects of this study. Attrition occurred for both the survey where 12 of the sample were excluded due to incomplete information (or because they did not fit criteria) and the interviews as four participants did not respond to the invitation to be interviewed and another four were unable to be scheduled appropriately. In sum, thirty practitioners (males *n* = 18, females *n* = 12) completed the qualitative study (see Table 1) which resulted in a 58% response rate for interview as this was based on the numbers that completed the initial survey.

**TABLE 1:** **Summary of the International Sample of Practitioner Sport Psychologists**.

**Location**	**Accrediting/Licensing Body**	**Total Accredited**	**Percentage of Available Sample (*n*)**	**Percentage of Sample**
North America	Association of Applied Sport Psychology (Certified Consultant)	334	3.89% (13)	43.33%
UK	Health Care Professionals Council (Practitioner Psychologist, Sport Psychology)	215	3.72% (8)	26.66%
Australia	Australian Psychological Society (College of Sport and Exercise Psychology)	221	2.26% (5)	16.66%
Ireland	Irish Institute of Sport (Registered Member)	25	16 % (4)	13.33%

### Materials

A semi-structured interview guide was developed for the purpose of this study. The question items were designed to elicit responses to a broad range of both positive and negative work-related experiences among a sample of applied sport psychologists. For instance, they were probed on their experiences of work engagement (e.g., Can you think of a specific time when you could describe yourself as engaged in your work?), burnout (e.g., Can you think of a time when you just weren’t able to recover from work?), and their use of social support as a personal resource (e.g., Can you think of a specific time when you turned to someone you trust in a time of stress?).

Combined with these prompts the interview employed a “critical incident technique” (CIT; Flanagan, 1954; Gremler, 2004) in order to encourage the participants to recall in rich detail, an experience that related to the area of investigation. CIT obtains a record of specific behaviors from those in the best position to make the required observations (Gremler, 2004). If the participant felt they had not experienced the behavior themselves, they were prompted to recall if they had witnessed a certain characteristic in others, however they were encouraged to use their own experienced above those of another.

### Procedure

Institutional ethical approval was granted for this study and subsequently, 80 practitioners who met the inclusion criteria for the study were identified, emailed, and invited to participate in the online survey. The recruitment email detailed the process of participation, with individuals invited to complete an online questionnaire to glean basic demographic information and confirmation that they were accredited as practitioners. After they completed the questionnaire, participants received a personal email thanking them for completing it and then they were sent an email outlining the next phase of the study.

Semi-structured interviews were organized, taking into account international time zones and the availability of both the participant and researcher, and conducted via Skype^TM^. The duration of the interviews ranged from 40 to 100 min and all were audio-recorded with consent from the participant. The interviews were then transcribed verbatim, and the transcripts were sent to each participant for approval.

### Analyses

Data analysis was conducted through NVivo V.10 software. The qualitative data analysis utilized a thematic analysis framework (Braun and Clarke, 2006), and proceeded in a number of steps. The researcher became familiar with the data through transcription and spent some time re-reading the script and re-listening to the audio recordings. Open coding determined the separation of the data initially; as the interviews were semi-structured, answers from different sections were able to be coded under the same heading at this point. Following the guidelines from Braun and Clarke (2006) initial codes were generated and line-by-line analysis was conducted in order to gather relevant data for each potential theme. The coded themes were isolated and more specific themes within each section were identified. By subjecting the data to an inductive analysis, the classification of the information and further reduction of the information into manageable units served to reflect both the reality of the participants and to shed light on their interpretation of their reality from their interviews (Braun and Clarke, 2006).

Each interview script was scrutinized for examples of burnout as defined by Maslach (2003, 2011) and subjectively assigned the attribute of either moderate to high levels of experienced burnout (high burnout), moderate to low levels of experienced burnout (low burnout) or experiencing no burnout (not-applicable).

Work engagement experiences were examined in all participants comparing level of experienced burnout. Finally, a frequency analysis was conducted to examine the extent to which those with high burnout versus low burnout recalled the use of social support and specifically where that support was sourced. The process of qualitative analysis employed was not rigid but was instead fluid and flexible in nature, so these steps followed in an interactive fashion. This was based on the approach of Braun and Clarke (2006) who stated that “analysis involves a constant moving back and forward between the entire data set, the coded extracts of data that you are analyzing, and the analysis of the data that you are producing” (p. 86).

## Results and Discussion

The findings are discussed under the primary themes of burnout, work engagement, and social support, followed by discussions, conclusions and suggestions for future research.

### Burnout

Each participant was subjectively categorized as either “high burnout” or “low burnout” or “non-applicable.” However, examples of burnout were readily reported by almost all participants. Similar to the findings of Dunford et al. (2012) emotional exhaustion was the most frequently cited dimension of burnout reported, as exemplified by P02/P12:

…And when we talk about depression and things, I actually think looking back on it now, and having had a year’s distance from it; it was not a positive experience in my life, I contemplated giving up, I was so unhappy in my position.

And it can cause emotional exhaustion because sometimes there aren’t things that you can do about it. If I’m frustrated with a coach or two …and then you get frustrated and then you question why do I even do this?…I don’t take it personally, but you know it’s frustrating because they don’t allow you to do your job the way it needs to be done and it creates emotional exhaustion and frustration and gets you to question whether or not you want to even be here anymore.

Feelings of emotional exhaustion were not limited to those who had experienced high levels of burnout. Even those in the low burnout group had episodic experiences of exhaustion, with one participant (P04) stating:

But at that time I do remember vividly having that feeling of…it was more the exhaustion mentally and physical and I just thought “I’ve nothing there, I can’t give you anything.”

In contrast to those who had experienced high levels of burnout, these latter reports related to less sustained experiences and a single case load. Once the competitive season finished, these feelings of exhaustion dissipated. For some participants, especially those who held a dual role in academia, the balancing of existing workload and their willingness to take on extra work to satisfy their own desires took a toll on their resources; P14 highlights this issue by saying:

I still loved doing the applied work [but] it was at that point just another thing that I had to get done versus something I really enjoyed and looked forward to because I was just trying to cram it in along with anything else. And so I’ve been really trying to work towards trying to chunk off time periods which are specifically devoted to my applied work, that way it sort of stays in that box and then my academic stuff stays within a different box …

Although instances of the following were less readily reported, participants from both high and low burnout out groups had also purportedly suffered from decreased feelings of efficacy, the dimension highlighted by Maslach et al. (2001) as being self-evaluative. It is highly plausible that the work being conducted was to a required standard, however feelings of incompetence and reduced productivity can negatively affect an individual’s sense of well-being. P21 highlights this issue in the following excerpt:

…well let’s see my, my performance was not good, I stopped doing things to the best that I could I started doing things just to get them done. So that was pretty difficult until another option was available. So I stopped being really proud of the work I was doing, which is never a good thing I always would be proud of what I’m doing rather than not.

As with work engagement, experiences of burnout were common and varied amongst the sport psychologists. What appears to differ between participants is the resources employed to combat the negative impact of burnout on their well-being. Two appear prevalent, the experience of work engagement as a buffer against burnout, and the utilization of social support as a social resource.

### Work Engagement

The practitioners were all able to recall instances of work engagement and verbalize what work engagement meant to them. Using a matrix coding query, we found that those who had not experienced high levels of burnout recalled instances of all three dimensions of work engagement at a different intensity than those who had experienced high levels of burnout. Statements regarding work engagement were coded as whether they represented vigor, absorption or dedication (see Table 2, for an explanation for each code). Dedication was identified most readily irrespective of their reported levels of burnout. As stated by P04:

**TABLE 2:** **Explanation of Coding Categories for Work Engagement**.

**Code**	**Child Node**	**Meaning**
Work Engagement		Any quotes that encapsulate the idea of Work Engagement
	Absorption	Individuals are fully concentrated and engrossed in their activities, time passes quickly and they find it hard to detach themselves from work (Hakanen and Schaufeli, 2012).
	Dedication	Strong involvement in one’s work accompanied by feelings of enthusiasm and significance and by a sense of pride and inspiration (Hakanen and Schaufeli, 2012).
	Vigour	High levels of energy and mental resilience while working. The willingness to invest effort in one’s work and the ability to avoid being easily fatigued. Persistence in the face of difficulties (Hakanen and Schaufeli, 2012).
	Flow	A sense that one’s skills are adequate to cope with the challenges at hand, in a goal-directed, rule bound action system that provides clear clues as to how well one is performing. Concentration is so intense that there is no attention left over to think about anything irrelevant, or to worry about problems. Self-consciousness disappears and the sense of time becomes distorted. People are willing to do an activity for its own sake, with little concern for what they will get out of it (Csikszentmihalyi, 1990).

Without question it’s when you see that light bulb moment in somebody’s eyes. When they have come to you wanting to be somewhere in their life in terms of their sports performance, or other areas in their life, because it wouldn’t be only sport that I deal with an athlete, there would be other issues that would come up. You’ll see somebody and you’ll know in their mind they want to be able to achieve something when they come to you but they usually are away from that, they’re not getting it for whatever reason, and then when I can identify what are the obstacles, and the barriers and the gaps that are in the way, and help them realise that they actually already have the tools to deal with it, and all I’m going to do is facilitate them in bringing them out. That Ping! Moment when they go “Ooooh I can do this!” … It’s just – I love that, I love that! It’s such a kick.

Both vigor and absorption were commonly reported, but more frequently by those who had not experienced high levels of burnout, compared to those who had experienced high levels of burnout. An example of absorption is as follows (P07):

I can relate to that especially when you’re working with people it’s difficult to have a concept of time because you’re so engaged. You have to be so in the moment to actually be present with the individual. You almost forget how much time has passed definitely when I’m working one-on-one with people and also when researching and reading I would feel the same thing for a lot of time has passed and not really conscious of that because I am very engaged in what I’m doing.

Similarly, P17 shares their experience of vigor during an episode of high engagement as having:

…High energy levels, kind of really excited about the challenge of doing what I was doing. I felt really motivated, which made me be really organised and plan out what I was doing and think about things thoroughly.

Although these quotes are only a small extract from the sample of recorded quotes, they clearly convey the different areas within which sport psychologists experience work engagement. Furthermore, they also reflect the degree to which practitioners are passionate about their work, how they garner meaning from working with athletes; seeing them grow as both performers and individuals.

### Social Support

A matrix coding query was conducted, looking at the frequency of each social support source for those who had experienced high and low levels of burnout. Our findings indicated that there appeared to be some differences in the magnitude with which individuals with low versus high experienced burnout utilized different sources of social support (see Table 3).

**TABLE 3:** **Explanation of Coding Categories for Social Support**.

**Code**	**Node**	**Child Node (i)**	**Child Node (ii)**	**Meaning**
Social Support				Any quotes that encapsulate the idea/ benefits of social support.
	Non- Work			Social support from outside the field of sport psychology.
		Family and Friends		Family and friends unrelated to sport psychology.
		Formal		This is where a participant has sought professional counseling or guidance outside of their organization.
		Peer		Alternatively people who work in their organization but are not within the same field i.e. other service providers.
	Work			Social support from within the field of sport psychology or formally from within the academic organization.
		Formal		
			Organizational	Where a participant cites using services provided by their organization i.e., an Employee Assistance Program (EAP).
			Peer	A formalised peer group within the organization.
			Supervisor	A person or person(s) who has been formally assigned to the participant to provide guidance in a managerial capacity. Their direct line manager, HOD, director of performance etc.
		Informal		
			Mentor	Someone within the organization who acts as an informal mentor to the participant.
				Or a previous mentor who the participant continues to seek for advice and guidance i.e., previous PhD supervisor.
			Peer	Friends or colleagues within the organization who the participant turns to in order to vent, etc., Or other Sport Psychologists who they collaborate with but who do not work in their organization.

The analysis revealed that participants identified several sources of social support, and for the purposes of our analysis, these were categorized and themed into work based versus non-work based. Firstly, work based sources of social support were divided into formal and informal sources of support. Examples of formal work support included support received from work-provided counseling, supervisors and superordinates, and formalized peer groups. Informal work support sources included informal mentors and informal peer groups. Non-work based sources of support included, for example, family and friends, personal counseling (e.g., if sought by the individual independently to their place of work) and peer support, which included colleagues who were explicitly identified as part of their organization but who did not bear the role of sport psychologist (e.g., other sport science and medical personnel).

In the findings, work based sources of social support were cited most frequently. Work based social support can contribute to an individual’s identification with an organization, especially for those who work virtually (i.e., working from home or on the road and outside the traditional centralized office; Wiesenfeld et al., 2001). The most notable source of formalized social support was support from a supervisor when one participant (P15) stated that:

It’s nice to be able to run things by somebody. I also have a supervisor who lives in a city interstate, and I can always just pick up the phone and ring him there and say: hey, I’ve got this problem what do you think?

Meta-analytic reviews have concluded that social support from a supervisor has been shown to decrease the perceived workload of an individual (Bowling et al., 2015). Their meta-analysis also showed a similar relationship between support from one’s co-workers and work stress. Participants reported using this source of social support in both formal and informal ways as P02 commented:

If you’re talking about peer support within an academic situation I’ve got people I turn to, [in the] research situation again, I’ve got people to turn to….I’ve had a number of, kind of formalised peer supervision groups for my applied work and I find them invaluable.

This quote is supported by another participant, P07, who states:

“[another practitioner] was in London during the games and like I didn’t meet up with him but we were in pretty regular contact we were - and it was informal but it was helpful because I knew, he knew what I was feeling you know and I remember that he sent me a couple of messages that were really kind of spot on and that was a very informal thing but it was, it was an outlet, and I knew, I think I did ring him once and we had a pretty long conversation yeah.”

Both types of peer related social support were cited as being instrumental to managing work stress, however informal peer support was more commonly cited. Those who had not experienced high levels of burnout reported both sources more readily than those who had experienced high levels of burnout.

Another form of social support commonly reported by the practitioners was that obtained from friends and/or family. Lapierre and Allen (2006) believe that both emotional support and instrumental sustenance (relieving family members of home based tasks or responsibilities) can alleviate work-family strain and contribute to employee well-being. One participant, P21 stated:

“I always say that my wife is fantastic and very supportive, and she knows when I get busy I’m going to do a little bit less of the work around the house, she’s going to help out with that…I would also say that socially on top of my wife, I have a group of friends, a very, very fond group of friends that are just a fantastic social support network. To the extent that if I ever say I need something, I will tell them I need something and they will do everything that they can to help.”

The above quote shows the influence of both friends and family on relieving work-family strain, which ultimately reduces an individual’s perceived work load thus reducing his or her work-related stress enhancing their well-being.

However, the most common sources of social support accessed by our sample seem to be informal in nature. Participants did not indicate that the support was organized or formalized, and was frequently sought on an *ad hoc* basis. Informal peer support was the second most highly cited form of support after family support. Those who had not experienced high burnout cited informal peer support more often than those who had experienced high levels of burnout. An example of informal support is provided by another participant, P24 who states:

I have been pretty fortunate for the eight years that I have been at the (organisation) because I have had a great group of sports psychologists and clinical psychologists that have been part of, that I have shared my work with. And that group has change quite significantly over the 8 years, but it has always been a big source of professional support for me.

Interestingly, those who were deemed to have experienced high levels of burnout cite family and friends more frequently than those who had experienced lower levels of burnout, with a 3:2 citation ratio for those who have experienced high levels versus those who had not. Family support often comes in the form of the ability to vent, or detach from work. As one participant P05 states:

So I think that of course there is my family, my brother, my father, my mother – I know I can always talk to them if there’s something annoying me or pissing me off about something, or that I’m stressed out about something.

And another practitioner, P21, comments:

But my wife and my friends, we escape work I mean when we’re together we very rarely talk about the work that we’re doing. But we’re doing fun activities you know whether it’s related to a youth sporting contest or you know I have a group of friends that one of us holds a get-together maybe once every second week, so we’re always together. So like we vacation together so you know it allows us to escape really.

Those who cited “friends” in the field of sport psychology were coded under “Work/informal/peer.” In sum, participants used social support as a resource, indicating the important role at hat this resource plays in these lives. However, support seemed rather unorganized, heavily based on family and friend support systems, with greater focus on short-term relief rather than long-term coping.

## Discussion

### Burnout

Participants in this study who combined their work in academic institutions as well as practicing applied sport psychology, often cited bringing work home, working from home, staying in the office late, or attending sporting events out of office hours, indicating that there might be little true psychological detachment from work. From a purely applied perspective, there were instances cited where the participants reported they were unable to detach from their work, especially when on site at camp or competition, which echoes the sentiment by Schein that a consultant is always consulting (as cited in Aoyagi and Portenga, 2010, p. 256). Despite the fulfillment of their job, all participants reported experiencing incidents of exhaustion at some point in their career, but only some reported full-blown experiences of burnout.

### Work Engagement

Engagement was frequently cited by our participants. When people are engaged in what they do, their well-being (both physical and psychological) will increase (Deci and Ryan, 2000). Hakanen and Schaufeli (2012) found that even though burnout can predict a person’s level of depression in the long term, the positive effect of work engagement on life satisfaction outweighs the negative effects of burnout. Those who had experienced high levels of burnout cited less frequently all dimensions of work engagement but were still able to recall times of work engagement and passion regarding their work. Therefore, it is invaluable to an individual’s work-related wellbeing to reduce instances of burnout and to support long-term work engagement

### Social Support

Burnout was experienced by the participants in this study. However, a resource that can both buffer the individual from burnout and foster work engagement is social support. According to Hobfoll’s (1989) conservation of resources theory, social support is a key resource that can preserve valued resources. In our study, we concluded that social support often facilitates recovery or can be the source of recovery, i.e., when an individual’s loved ones will encourage them to take time off and detach from work.

Support from supervisors has also been found not only to protect work engagement during times of high stress, but to be a key resources in encouraging work engagement in employees (Bakker et al., 2007). Furthermore, Schaufeli and Bakker (2004) also found evidence for a positive relationship between social support (both support from colleagues and supervisory coaching) and work engagement. On the other hand, job resources had a negative effect on burnout, meaning that the greater the resources, the less likely an individual is to burn out.

Notably, our sample highlighted the frequent use of informal forms of social support and rarely reported using formal forms of social support. Prior research has suggested that noted that a main reason for not seeking professional help in terms of personal psychotherapy is the perceived difficulty in finding an acceptable therapist (Bearse et al., 2013). While this may be a contributing factor to the more commonly reported reliance on informal social support, it is also possible that due to individualized nature of the work of many practitioners, having formalized social support is less available. Rhodius and Sugarman (2014) recommends establishing peer support groups to combat this potential isolation but notes that it is not required by an accrediting body other than during supervised training, with the exception of the Australian accreditation process for practicing psychologists. Interestingly, Cogan et al. (2012) highlighted the benefits of working in teams during the provision of psychological support to athletes at the Olympic games. This formal peer-support structure was unique and it may be a useful way to manage risk among practitioners during periods of intensive workload.

Future research could investigate the challenges of practitioners staving off burnout by targeting the formal peer support networks and exploring the role of social support, boundary issues and specific self-care behaviors, which may include what Walsh (2011) has termed “therapeutic lifestyle changes.” These lifestyle changes proposed by Walsh (2011) are each linked to mental health benefits and include (effective nutrition, exercise, and physical activity). Other possibilities for future research include mindfulness interventions for practitioners. They could be implemented into practitioners daily life for self-care in addition to their use to enhance the consultation preparation process. A recent study reported that sport psychology practitioners tend to use mindfulness activities within consultation sessions, but not for their own self-care as practitioners (McAlarnen, 2015). The challenges for practitioners in dealing with diverse athlete groups may present a challenge which requires further scrutiny. Another interesting and fruitful avenue for future research would be to specifically longitudinally investigate well-being in a select cohort of applied sport psychologists over a concerted block of time. This block of time optimally would coincide with a major sporting event (e.g., Olympic Games, world championships) which would see this group of practitioners work prior to, during, and after this major event with teams and individuals. The benefits of investigating sport psychologists well-being in the lead-up to, during and after a major sporting event will shed light on the unique demands placed on applied sport psychologists. Ultimately this type of research may lead to practitioner specific guidelines and recommendations regarding the promotion of appropriate self-care.

## Conclusion

This study has provided tentative evidence for the role of social support in ameliorating burnout and other challenges to mental health in a sample of applied psychology practitioners. Instead, positive work engagement was integral to the practitioners experiences and the role of formal support networks may be a key feature to consider (Cogan et al., 2012). It is arguable that the social context rather than individual competencies were a key consideration in the development of psychological resources. Applied sport psychologists act as support to many athletes and sports professionals, often instructing them to live balanced lifestyles and encouraging them to build a strong and supportive network around them, yet according to our study this is not always the case with the practitioners themselves. Burnout appears to creep into this profession regardless of the high rate of work engagement. Hakanen and Schaufeli (2012) found that even though burnout can predict a person’s level of depression in the long term, the positive effect of work engagement on life satisfaction outweighs the negative effects of burnout. Therefore, it is valuable to not only reduce instances of burnout but to support and encourage long-term work engagement.

Our research adds to the accumulating evidence and notion that mental health and indeed the viability of applied psychology rests not just on the achievement of successful outcomes for clients but—also—on the ongoing mental health of practitioners. Furthermore, future research can build on our findings in exploring the optimal use of psychological and social resources in reducing chances of burnout of applied psychologists.

### Conflict of Interest Statement

The authors declare that the research was conducted in the absence of any commercial or financial relationships that could be construed as a potential conflict of interest.
